# IQGAP1 and IQGAP3 are critical host factors for Marburg virus replication, nucleocapsid transport, and cell-to-cell spread

**DOI:** 10.1007/s00018-025-06047-3

**Published:** 2026-02-09

**Authors:** Olga Dolnik, Kathleen Voigt, Victoria Hunszinger, Cornelius Rohde, Susanne Berghöfer, Martin Schauflinger, Andreas Rausch, Thomas Schanze, Stephan Becker

**Affiliations:** 1 Institut für Virologie, Philipps-Universität Marburg, Marburg, 35043 Germany; 2 Institute of Biomedical Engineering, THM University of Applied Sciences, Giessen, 35390 Germany; 3 German Center for Infection Research (DZIF), Partner Site Giessen-Marburg-Langen, Philipps-Universität Marburg, Marburg, 35043 Germany; 4 Nanoinfektionsbiologie, HZI - Helmholtz-Zentrum für Infektionsforschung GmbH, Braunschweig, 38124 Germany; 5 Institut für Molekulare Virologie, Universitätsklinikum Ulm, Ulm, 89081 Germany; 6 Oculus Optikgeräte GmbH, Wetzlar, 35582 Germany

**Keywords:** IQGAP1, IQGAP2, IQGAP3, Marburg virus, Actin, Virus spread, Filopodia

## Abstract

The IQGAP protein family—comprising IQGAP1, IQGAP2, and IQGAP3—exhibits structural similarity but fulfils distinct cellular functions. We previously demonstrated that IQGAP1 is recruited to Marburg virus (MARV)-induced inclusion bodies (IBs) and associates with motile nucleocapsids. To further elucidate the roles of IQGAP proteins in the MARV life cycle, we generated Huh-7 cell lines with single, combined, or triple knockouts (KOs) of IQGAP isoforms. Loss of IQGAP proteins consistently reduced cellular permissiveness to MARV infection and impaired multiple key viral processes: (i) transcription and replication efficiency was diminished predominantly by IQGAP3 KO; (ii) virus release was most notably reduced in IQGAP3 KO cells, whereas cell-to-cell spread was more strongly impaired in IQGAP1 KO cells; and (iii) although actin tails continued to form at nucleocapsids in triple KO cells, long distance nucleocapsid transport was altered, with reduced spatial displacement efficiency observed in both IQGAP1 KO and IQGAP3 KO cells. The expression of individual IQGAPs in triple KO cells demonstrated their functionality and ability to partially restore the phenotype of wild-type cells. These findings identify IQGAPs as critical host factors that support MARV transcription/replication, nucleocapsid transport, and viral spread, likely through modulation of actin dynamics.

**Supplementary Information:**

The online version contains supplementary material available at 10.1007/s00018-025-06047-3.

## Introduction

The genus *Orthomarburgvirus* within the family of *Filoviridae* includes highly pathogenic zoonotic viruses that cause re-emerging outbreaks in humans in sub-Saharan Africa, posing an ongoing threat to public health [1]. The viral RNA genome of the species *Orthomarburgvirus marburgense* (MARV) is encapsidated by the nucleoprotein NP, which together with four additional viral proteins (VP) forms the nucleocapsid (NC): the polymerase L, VP35, a cofactor of the polymerase, the transcription factor VP30 and VP24, whose function is so far not well investigated for MARV. However, the homolog of VP24 in the closely related Ebola virus has been shown to be an essential nucleocapsid condensation and transport factor with an anti-IFN signaling function [2, 3]. The MARV matrix protein VP40 surrounds the nucleocapsids, forming a regular layer beneath the viral envelope into which the glycoprotein GP is incorporated [4, 5].

Filovirus entry into target cells is initiated after attachment of GP to the plasma membrane and is accomplished by macropinocytosis [6–8]. During maturation of macropinocytic vesicles into late endosomes/lysosomes (LE/Lys), the viral envelope fuses with the LE/Lys membrane, and the NC is released into the cytosol, where it serves as a template for the primary transcription of capped viral mRNAs [9, 10]. Translation of viral proteins occurs at free ribosomes, with the exception of GP, which is translated at the rough endoplasmatic reticulum. GP is transported through the classical secretory pathway via the Golgi apparatus to the plasma membrane [11]. The matrix protein VP40 associates with membrane vesicles and is also transported to the plasma membrane where budding is induced, to form the viral envelope together with GP and cellular lipids where it drives together with nucleocapsids particle assembly and budding [12].

Expression of the nucleocapsid proteins (NP, VP35, VP30, VP24 and L) leads to the formation of inclusion bodies (IBs) where secondary transcription, genome replication and *de novo* nucleocapsid assembly are organized, reviewed in [13, 14]. Nucleocapsids are transported directionally from IBs to budding sites through actin polymerization-driven transport mediated by actin tail formation at one end of the particles [15, 16].

Isoleucine Glutamine motif-containing GTPase activating proteins (IQGAPs) are a family of evolutionary conserved scaffold proteins that interact with many partners to regulate various cellular processes, including cytokinesis, migration, proliferation, intracellular signalling, vesicle trafficking and cytoskeletal dynamics, reviewed in [17–21]. In human cells three isoforms, IQGAP1, IQGAP2 and IQGAP3 are expressed with highly homologous functional domains, reviewed in [22, 23]. IQGAP1 is ubiquitously expressed and represents the best-characterized isoform, whereas IQGAP2 exhibits a more organ-specific expression pattern, with the highest levels in the liver and prostate [24]. IQGAP3 is also broadly expressed, showing the highest expression in the brain and testis [25]. The precise functions of these isoforms are still under active investigation. Advances in cancer research revealed significant importance of all IQGAP isoforms in oncogenesis and point to their divergent functionality despite of high homology at protein level [26–31]. It has been shown that IQGAP1 and IQGAP3 possess pro-oncogenic functions and high intracellular levels represent poor clinical prognostic markers [32–36]. IQGAP2, however, represents a tumour-suppressor and its expression is reduced in most cancer cells [27, 30, 37, 38].

Proteins within the IQGAP family have been recognized for their involvement in infectious processes. For instance, the role of IQGAP1 in the invasion and pathogenesis of bacterial pathogens has been documented across several studies [39–42]. Furthermore, IQGAP1 has been found to interact with several viral proteins, predominantly facilitating pro-viral functions that support viral egress. Conversely, Sabo et al. [43] identified an antiviral role for IQGAP1, where its interaction with the nucleoprotein of human immunodeficiency virus type 1 (HIV-1) and the p6 domain of gag inhibited the association of the HIV-1 capsid with the plasma membrane. In the context of HCV-infected hepatocytes, IQGAP2 has been characterized as an antiviral effector of interferon-alpha [44]. Concurrently, IQGAP proteins are targeted by viral infections; notably, HBV infection leads to the SUMOylation and subsequent degradation of IQGAP2. This degradation process is linked to the downregulation of SNP3, a protease responsible for the de-conjugation of SUMO proteins. The reduction in IQGAP2 levels mitigates the inhibition of Akt phosphorylation, thereby enhancing host protein translation and concurrently diminishing HBV protein expression [45]. A genome-wide screen of human papilloma virus-infected cervical epithelial cells revealed upregulation of IQGAP3 mRNA [46]. These examples underscore the significance of IQGAP family proteins as pivotal regulators in the viral infection process, exhibiting diverse functions across a range of viruses.

The human pathogenic filoviruses MARV and Ebola virus (EBOV) exploit proteins active in cytoskeleton dynamics [12, 47]. For example, contrail-like actin tail formation at EBOV nucleocapsids that drives their transport is dependent on Arp2/3, Rac1 and WAVE1 [48]. In addition, we detected IQGAP1 in these contrail-like structures at one end of moving nucleocapsids. Moreover, IQGAP1 is recruited into the viral-induced inclusion bodies in the cytosol of MARV-infected cells [49]. Down-regulation of IQGAP1 in MARV-infected cells resulted in reduction of virus release. In addition, a mutant virus deficient in recruitment of IQGAP1 into IBs and to nucleocapsids displayed changed IB morphology, nucleocapsid motility and reduced budding from filopodia and formed smaller plaque size in cell culture [49]. It was, however, not clear whether IQGAP1, which is recruited to the MARV nucleocapsids is essential for the formation of the contrail-like actin tails. In addition, the function of IQGAP1 in the viral inclusion bodies and during viral entry was enigmatic, as was the role of other IQGAP isoforms.

Here we analysed the role of the IQGAP protein family members in MARV infection using IQGAP knockout (KO) cell clones. We found that IQGAPs support MARV propagation at several steps of the viral life cycle. Knockout of IQGAP1 impaired mainly nucleocapsid transport and formation of viral foci. Additionally, we could show that IQGAP3 KO displayed strong effects on viral transcription/replication, nucleocapsid transport and virus release. Expressing individual GFP-tagged IQGAPs in triple IQGAP KO cells showed that all proteins were functional and could restore wild type phenotypes.

## Materials and methods

### Cells, viruses and antibodies

Human hepatoma cells (Huh-7) and African green monkey kidney cells (Vero E6) were maintained in Dulbecco´s modified Eagle medium (DMEM) supplemented with 10% fetal calf serum, L-glutamine and penicillin-streptomycin solution (Gibco, Karlsruhe, Germany). The Musoke strain of MARV (GenBank accession number DQ217792), the recombinant MARV-30RFP (rMARV-VP30RFP) [49] and recombinant MARV-VP30GFP were propagated on Vero E6 cells grown in DMEM supplemented with 3% fetal calf serum, L-glutamine and penicillin-streptomycin solution. Infections with MARV were performed under BSL-4 conditions at the Institute of Virology, Philipps University Marburg according to national regulations (IVMr46-53r30.03.UMR122.11.01).

Mouse monoclonal antibody anti-IQGAP1 (clone AF4, Upstate^®^) was purchased from EMD Millipore and a rabbit monoclonal antibody anti-IQGAP1 (D8K4X) was purchased from Cell Signalling. IQGAP2 was detected by a mouse monoclonal antibody (A-4) from Santa Cruz Biotechnology. The anti-IQGAP3 antibody was kindly provided by the laboratory of Professor Kozo Kaibuchi from Nagoya University, Japan. Mouse monoclonal antibody anti-GAPDH (6C5) was from Thermo Fischer. Phalloidin-TRITC from Sigma-Aldrich was used to label actin. Secondary goat anti-mouse HRP-conjugated antibodies were purchased from Dako and swine anti-rabbit HRP-conjugated antibodies from Dianova.

### Transfection and plasmids

Transient transfections of Huh-7 cells were carried out using TransIT-LT1 from Mirus according to supplier`s instructions. For actin labelling in living cells, pTaq-RFP-Actin from Evrogen was used. pGFP-IQGAP1, pGFP-IQGAP2 and pGFP-IQGAP3 expression plasmids were kindly provided by the laboratory of professor Kaibuchi from the Nagoya University, Japan.

### Cloning and rescue of rMARV-VP30GFP

Recombinant MARV with an additional ORF coding for VP30GFP (rMARV-VP30GFP) cloned into an artificial AvrII restriction site between the VP35 and VP40 genes was constructed as described earlier [15]. The rescue of the recombinant virus was performed as described by Dolnik et al., 2014 and sequencing of the viral RNA revealed no additional mutations to the reported silent mutations [49].

### CRISPR/Cas9 knockout of iqgaps

Addgene plasmid #62,988 (pSpCas9-2 A-Puro) was used to clone single guide RNAs (sg) to target IQGAP1 or IQGAP3 genes to achieve single functional knockout. The addgene plasmid pX333 (#64073) was used to generate the double IQGAP1 + 3_KO_ clone and triple IQGAPall_KO_ clone targeting all three IQGAP genes. For clone selection, the puromycin resistance gene was cloned into the pX333 plasmid between the restriction sites FseI and NotI.

sg sequences: IQGAP1 (NM_3870): 5´-TGAAAGGAGACGTCAGAACG-3`; IQGAP2 (NM_006633): 5´-AGTATTTGTCAGCACACTCC-3`; IQGAP3 (NM_178229): 5´-CACCGGGATCAACAAAGCCATCCGG-3´.

Huh-7 cells were transfected with plasmids coding for the indicated sg RNA and selection medium containing 2 µg/ml puromycin was added one day after transfection for 5 days. Limiting dilution was then used to seed one cell/well into 96 wells plates and grow the KO cell clones.

### SDS-PAGE and Western blot analysis

An 8% (for IQGAP) and a 12% (for viral proteins) SDS-PAGE and Western Blot analysis were performed as previously described [50, 51]. Briefly, cells and culture supernatants were diluted with sample buffer (100 mM Tris-HCl, pH 6.8; 0.2% bromophenol blue; 20% glycerol; 10% 2-mercaptoethanol; 4% SDS) and boiled for 10 min. After electrophoresis, proteins were blotted onto PVDF membranes (0.2 Cytiva Marlborough, USA) by semi-dry technique. Membranes were blocked with 10% milk powder and incubated for 1 h at room temperature with primary antibodies diluted in PBS/0.1% Tween 20 (PBS/T) with 1% milk powder. For the detection of IQGAP proteins, the mouse anti-IQGAP1 and the mouse anti-IQGAP2 antibodies were diluted 1:250 and the rabbit anti-IQGAP3 antibody 1:1000. MARV NP and VP40 were detected with mouse monoclonal antibodies diluted 1:1000. The GAPDH antibody was used at a dilution of 1 µg/ml. The membranes were then washed three times with PBS/T and incubated 1 h at room temperature with corresponding secondary antibodies diluted in PBS/T with 1% milk powder. A goat anti-mouse-HRP conjugated antibody at a dilution of 1:40.000 and a pig anti-rabbit-HRP conjugated antibody at a dilution of 1:20.000 were used as secondary antibodies. After washing with PBS/T and PBS, the proteins were detected by chemiluminescence substrate (SuperSignal™; Thermo Fischer, Waltham, USA). The signal intensity was quantified using Chemidoc system and the ImageLab software from Biorad.

### Live cell imaging and particle tracking

Live cell microscopy of cells infected with rMARV-VP30RFP or rMARV-VP30GFP was performed as described previously [15, 49]. Briefly, cells were seeded into 8 well µ-slide (Ibidi, Munich) 24 h prior to infection. Cells were infected at an MOI of 0.1 plaque forming unites per cell for 1 h in Opti-MEM lacking phenol red (Life Technologies). Next, the inoculum was removed and cells were transfected with one of the plasmids encoding the different GFP-IQGAPs or Tag-RFP-actin in CO_2_-independent Leibovitz´s medium. Time-lapse experiments with living cells were recorded with a Leica DMI6000B using a 63x oil objective. Images and movie sequences were processed with Leica LAS X software. The movement of viral NCs was analyzed using IMARIS software version 10.0.1 (Oxford Instruments). NCs were identified using the *Spots* detection algorithm in IMARIS (Bitplane, Oxford Instruments), with the estimated spot diameter set to 1 μm. To distinguish the comparatively lower-intensity VP30-GFP signal associated with NCs from the highly fluorescent signal derived from VP30-GFP accumulation in inclusion bodies (IBs), the *Low fluorescence* intensity threshold setting was applied. For temporal analysis, NC trajectories were calculated using the autoregressive motion tracking algorithm. Tracking parameters were defined by a maximum displacement of 1 μm between consecutive time points and a maximum gap size of 3 frames, allowing for short-term signal interruptions during image acquisition.

### Immunofluorescence analysis and confocal laser scanning microscopy

Immunofluorescence analysis was performed as previously described [52]. Briefly, Huh-7 cells on cover slips were fixed with 4% PFA for 30 min at indicated time points after infection. MARV-infected cells were fixed overnight and then transferred into a fresh plate with new 4% PFA before being removed from the BSL-4 laboratory and further incubated overnight. PFA was removed and free aldehyde groups were quenched using 100 mM glycine in PBS. Afterwards, samples were washed with PBS and cells permeabilized with 0.1% Triton X-100 in PBS. The cells were incubated in blocking solution (2% bovine serum albumin, 0.2% Tween 20, 5% glycerol and 0.05% sodium azide in PBS) and stained with specific antibodies and phalloidin-TRITC or left unstained. Cover slips were mounted on glass cover slides with Fluorsave from Sigma Aldrich. After drying, they were examined using confocal laser scanning microscopy with either a Leica SP5 or a Stellaris microscope.

### Pearson’s correlation analysis with ImageJ

Confocal images were analysed in ImageJ using the pixel based Coloc 2 plugin to quantify the colocalization of IQGAP proteins with IBs. Colocalization of IQGAP proteins with inclusion bodies was evaluated using Pearson´s correlation coefficient (R-value) with Coste´s automatic threshold regression [53]. R-values range from − 1, indicating complete exclusion of colocalization, to + 1 with representing perfect colocalization of signals from both channels.

### Quantification of filopodia using filoquant

Confocal images of cells stained for actin were analyzed to determine the length and density of filopodia using the FiloQuant plugin in ImageJ [54]. Single-image and Semi-automated FiloQuant analyses were performed to quantify the number and length of filopodia. Filopodia density was calculated as the ratio of the number of filopodia per µm of cell edge.

### Minigenome assay

Minigenome assays were performed as previously described [55]. Briefly, the Huh-7 IQGAP KO cell clones were grown in 6-well plates and transfected in triplicates with plasmids encoding the MARV nucleocapsid proteins (0.5 µg pCAGGS-NP, 0.1 µg pCAGGS-VP35, 0.1 µg pCAGGS-VP30 and 1 µg pCAGGS-L) and 0.5 µg pCAGGS-T7, which encodes the T7 polymerase, and 1 µg p3M-5 M-R-Luc, which encodes the MARV-specific minigenome. In addition, 0.1 µg pGL4, which expresses firefly luciferase (Promega, Madison, USA) under the control of an SV40 promoter, was transfected to control cellular transcription efficiency. For the reconstitution of IQGAP activity in the KO cell clones either GFP or one of the GFP-IQGAP-encoding plasmids were transfected. At 48 h post-transfection, cells were lysed with passive lysis buffer (Promega, Madison WI, USA) and the *renilla* and *firefly* luciferase activities were measured using Renilla and Firefly juice kits (pjk, Kleinblittersdorf, Germany) on a luminometer (Berthold, Germany).

### Quantitative real-time PCR (qRT-PCR)

Quantification of viral RNA in MARV infected cells was performed using a one-step qRT-PCR as published by Panning et al. [56]. Total RNA was isolated from MARV infected control and IQGAP KO cell clones using the QiaAmp viral RNA kit (Qiagen GmbH, Hilden, Germany). Serial dilutions (10^2^−10^5^ copies per reaction) of MARV L-gene-specific transcripts were used as standard to calculate the RNA copies/ng total RNA of the samples. L gene-specific primers and probes were obtained from TIB Molbiol Syntheselabor GmbH (Berlin, Germany), and the reactions were performed using the Luna Probe One-Step RT-qPCR 4x Mix with UDG (New England Biolabs GmbH, Frankfurt, Germany).

### Focus-forming assay

Cells were inoculated in 12-well plates with two-fold serial dilutions of rMARV-VP30GFP and overlaid 1 h p.i. with 1.2% Avicel™ in MEM with 3% FCS. On day 3 p.i., the overlay was removed and cells were fixed with 4% paraformaldehyde (PFA) in DMEM for 48 h at 4 °C. Fluorescent images were then acquired with a 10x objective at a Nikon TS-100 microscope with a digital DS-5Mc camera and focus areas were determined using Fiji software. Mean focus size (> 100 foci) of control cells was set to 100% and compared to the mean focus size of KO cell clones.

### TCID50 assay

TCID50 assay was performed as described previously [49]. Vero E6 cells were cultured in 96-well plates and infected with 10-fold or 5-fold serial dilutions (eight replicates per dilution) of supernatants from MARV-infected cells. At 10 days p.i., when the CPE had been established, cells were examined by light microscopy. The TCID50/ml titers were calculated using the Spearman-Kärber method [57] and normalized to the amount of infected cells determined by immunofluorescence staining of the nucleoprotein.

### Statistical analysis

The data are presented as mean values with standard deviations from at least three independent experiments. Statistically significant differences between the cell clones were determined using GraphPad Prism with a one-way ANOVA followed by Tukey’s multiple comparisons test. Adjusted p-values were considered not significant (ns) for p ≥ 0.05, and significant for p ≤ 0.05 (*),p ≤ 0.01 (**),p ≤ 0.001 (***), and p ≤ 0.0001 (****).

## Results

### Subcellular localization of IQGAPs in MARV-infected cells

**Fig. 1:**
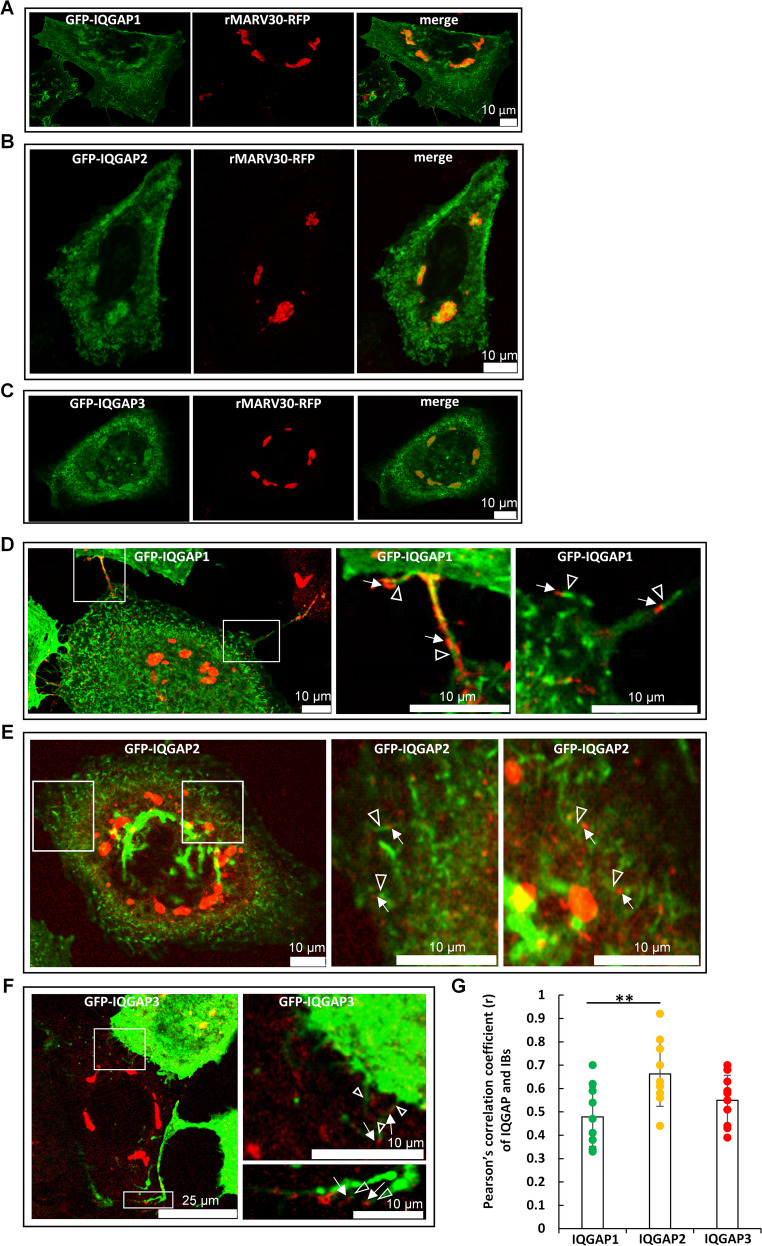
Localization of GFP-IQGAP isoforms in MARV-infected Huh-7 cells. Huh-7 cells were infected with recombinant MARV expressing VP30-RFP for nucleocapsid labelling and subsequently transfected with expression plasmids for GFP-IQGAP1, GFP-IQGAP2 or GFP-IQGAP3, respectively. Cells were fixed 24 h p.t. and subjected for confocal laser scanning microscopy at a SP5 Leica microscope. Scale bars are indicated in the merged images. **A-C.** GFP-IQGAP1, GFP-IQGAP2 and GFP-IQGAP3 are located in IB formed by MARV NP and labelled by VP30-RFP. **D.** GFP-IQGAP1 signal in green (see white arrowheads) at the rear of red nucleocapsids (white arrows) in protrusions contacting neighbouring cells and at the tip of protrusions. **E.** GFP-IQGAP2 signal in green (see white arrowheads) on nucleocapsids in red (white arrows) in the cytosol. **F.** GFP-IQGAP3 signal in green (see white arrowheads) at nucleocapsids in red (white arrow) at the tip of cellular protrusion contacting neighbouring cells. In images D-F, brightness was increased to better visualise the nucleocapsids. **G.** Localisation of the IQGAP proteins within IBs was evaluated using Pearson’s correlation analysis and is represented as the R-value of 10 inclusion bodies from at least 5 different cells across two independent experiments. Statistical significance was assessed using Tukey´s multiple comparisons test with an adjusted p-value **p ≤ 0,01

To evaluate the role of IQGAP family proteins in MARV infection, we expressed GFP-tagged constructs of all three human IQGAPs in MARV-infected Huh-7 cells and analysed their subcellular localisation (Fig. 1). As expected from our previous results, GFP-IQGAP1 was detected in IBs (Fig. 1A) [49], as were GFP-IQGAP2 (Fig. 1B) and GFP-IQGAP3 (Fig. 1C). Quantification revealed that approximately 70% of the IBs per cell were IQGAP-positive (66% ±29% for IQGAP1, 76% ±19% for IQGAP2, and 67% ±22% for IQGAP3).

Using Pearson´s correlation analysis of IBs showing IQGAP signals, we detected a moderate to high correlation between IQGAP proteins and MARV IBs (Fig. 1G). Furthermore, staining of endogenous IQGAPs recapitulated the localization patterns observed with the GFP–IQGAP constructs (supplemetary Fig. S1A–D).

All GFP-tagged IQGAPs were observed as contrail-like condensates (Fig. 1D and F, arrowheads) at one end of approximately 1 μm long VP30-RFP-positive particles (arrows) in the cytosol outside of viral IBs. Those particles have previously been shown to represent viral nucleocapsids [15]. Interestingly, thin GFP-IQGAP1-positive plasma membrane protrusions connecting neighbouring cells were observed to contain nucleocapsids with GFP-IQGAP1 condensates at one end (Fig. 1D). This phenomenon could also be observed with GFP-IQGAP2 and − 3 (Fig. 1E and F). Supporting these findings in fixed cells, live-cell time-lapse analyses revealed formation of GFP-positive contrail-like structures of all three fluorescently-tagged IQGAPs at the rear end of nucleocapsids moving in the cytosol (supplementary data, movies 1, 2, 3). These results suggested that IQGAP family proteins have specific functions during several steps of the MARV infection cycle.

### Generation and characterization of Huh-7 IQGAP KO cell clones

To explore the functions of the three IQGAP family members in MARV infection, we generated Huh-7 cells with single knockouts (KO) for IQGAP1 (IQGAP1_KO_) and IQGAP3 (IQGAP3_KO_), a double KO for IQGAP1 and 3 (IQGAP1 + 3_KO_) and a triple KO (IQGAPall_KO_) for all three IQGAP family members using the CRISPR/Cas9 technology (see overview Table 1). The specific homozygous mutations in the obtained KO cell clones were confirmed by Sanger sequencing. Western blot analyses finally confirmed the absence of the targeted IQGAP isoforms (supplementray Fig. S2 A). In contrast, all IQGAP isoforms could be detected in the Huh-7 control cell clone [no single guide (sg) RNAs transfected].

**Table 1.**
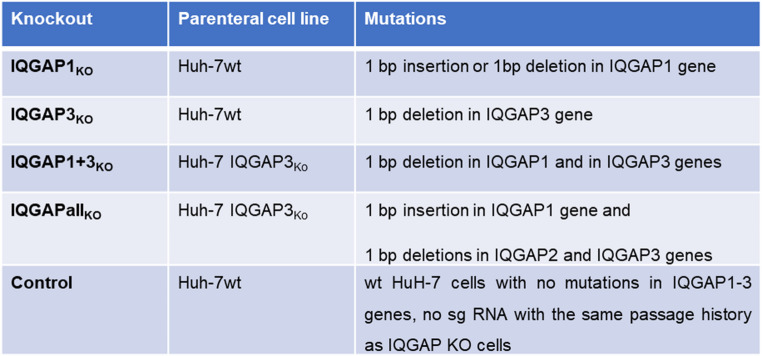
Huh-7 IQGAP KO cell clones generated by CRISPR/Cas9 method

### Effects of IQGAP KO on the actin cytoskeleton

Because IQGAP proteins are important players in the field of actin dynamics, we analysed the effect of the IQGAP KO on the intracellular distribution of actin by confocal microscopy. In addition, the length and density of filopodia in IQGAP KO cell clones was quantitatively assessed using the FiloQuant plugin of ImageJ [54].

**Fig. 2:**
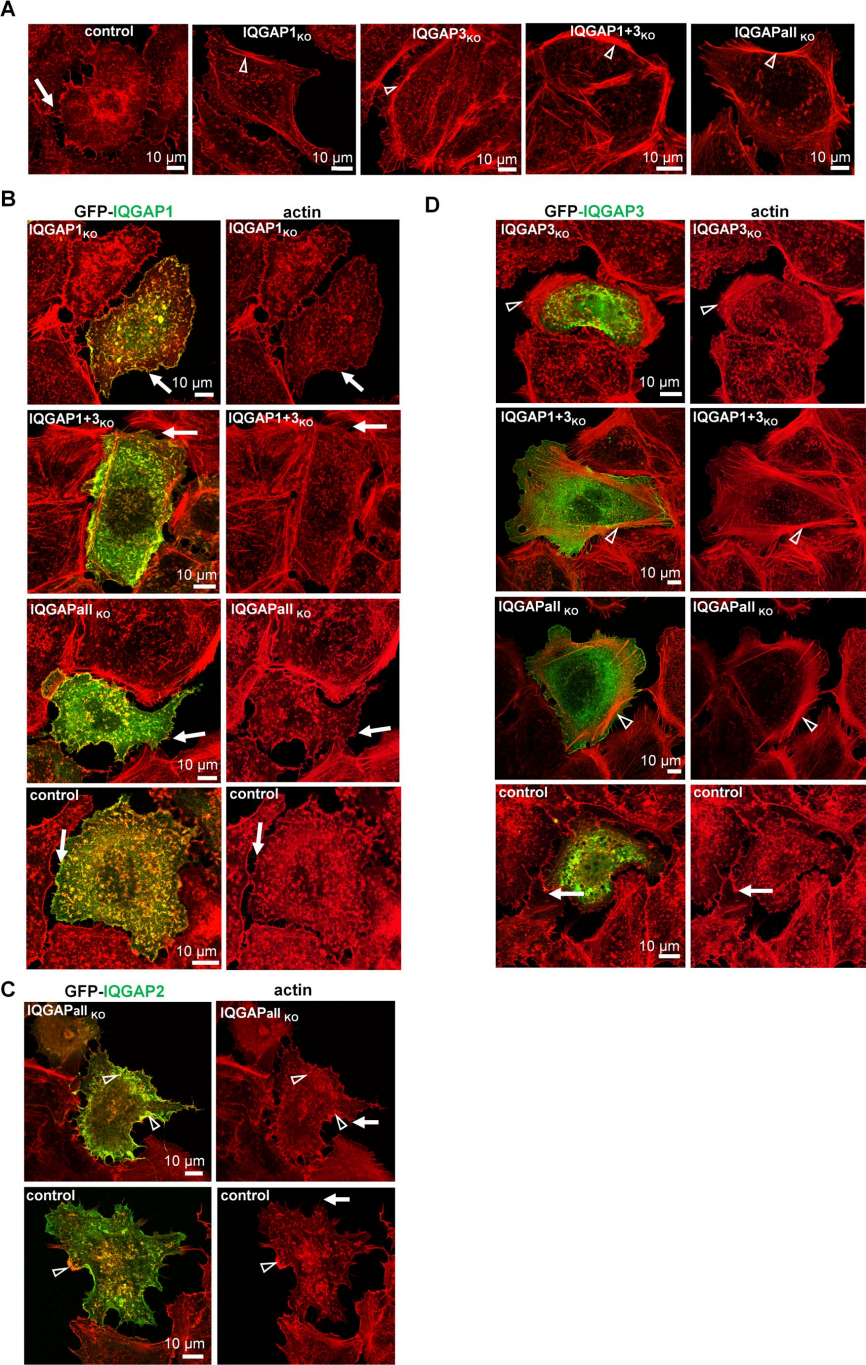
Characterization of the Huh-7 IQGAP KO cell clones actin phenotypes. **A.-D.** Actin cytoskeleton rearrangement in IQGAP KO cell clones after GFP-IQGAP isoform back-transfection. Indicated IQGAP KO cell clones and control cells were mock transfected **(A.)** or transfected with expression plasmids encoding either GFP-IQGAP1 **(B.)**, GFP-IQGAP2 **(C.)** or GFP-IQGAP3 **(D.)** and fixed 24 h p.t. Cells were then stained with Phalloid-TRITC and subjected to confocal microscopy analysis using a Leica SP5 confocal microscope. The white arrows indicate cell borders with filopodia and white arrowheads actin stress fibres or rearranged actin rich areas

All four IQGAP KO cell clones displayed actin rearrangements characterized by the appearance of prominent stress fibres in comparison to the control Huh-7 cell clone (Fig. 2A, white arrowheads). While the control cell clone apparently showed many filopodia and microspikes (Fig. 2A, white arrows), those seemed to be reduced in all IQGAPKO cell clones (Fig. 2A).

To evaluate whether the observed changes of the actin cytoskeleton could be attributed to the KO of IQGAP proteins, GFP-tagged IQGAP expression constructs were transfected into the respective KO cell lines. Expression of GFP-IQGAP1 into the all IQGAP1 KO clones restored the control phenotype, with less stress fibres (Fig. 2B, white arrows in GFP-IQGAP1-positive cells and in control cells). The ectopic expression of GFP-IQGAP2 into the IQGAPall_KO_ cells induced rearrangement of peripheral actin filaments into actin-rich membrane ruffles (Fig. 2C, white arrowheads), a phenomenon which has previously been reported upon IQGAP2 overexpression [58]. The expression of GFP-IQGAP3 in the corresponding KO cells (IQGAP3_KO_, IQGAP1 + 3_KO_ and IQGAPall_KO_) did not visibly reduce stress fibre formation (Fig. 2D, white arrowheads).

To validate the observations, we quantified the density and length of filopodia in the IQGAP KO clones using the FiloQuant plugin of ImageJ. This showed the reduction of filopodia density to be significant for the IQGAPall_KO_ clone (supplementray Fig. S2 B), the reduction of filopodia density in the other three cell clones missed statistical significance (Table 2). Filopodia length was not significantly different among all KO clones (Table 2).

**Table 2.**
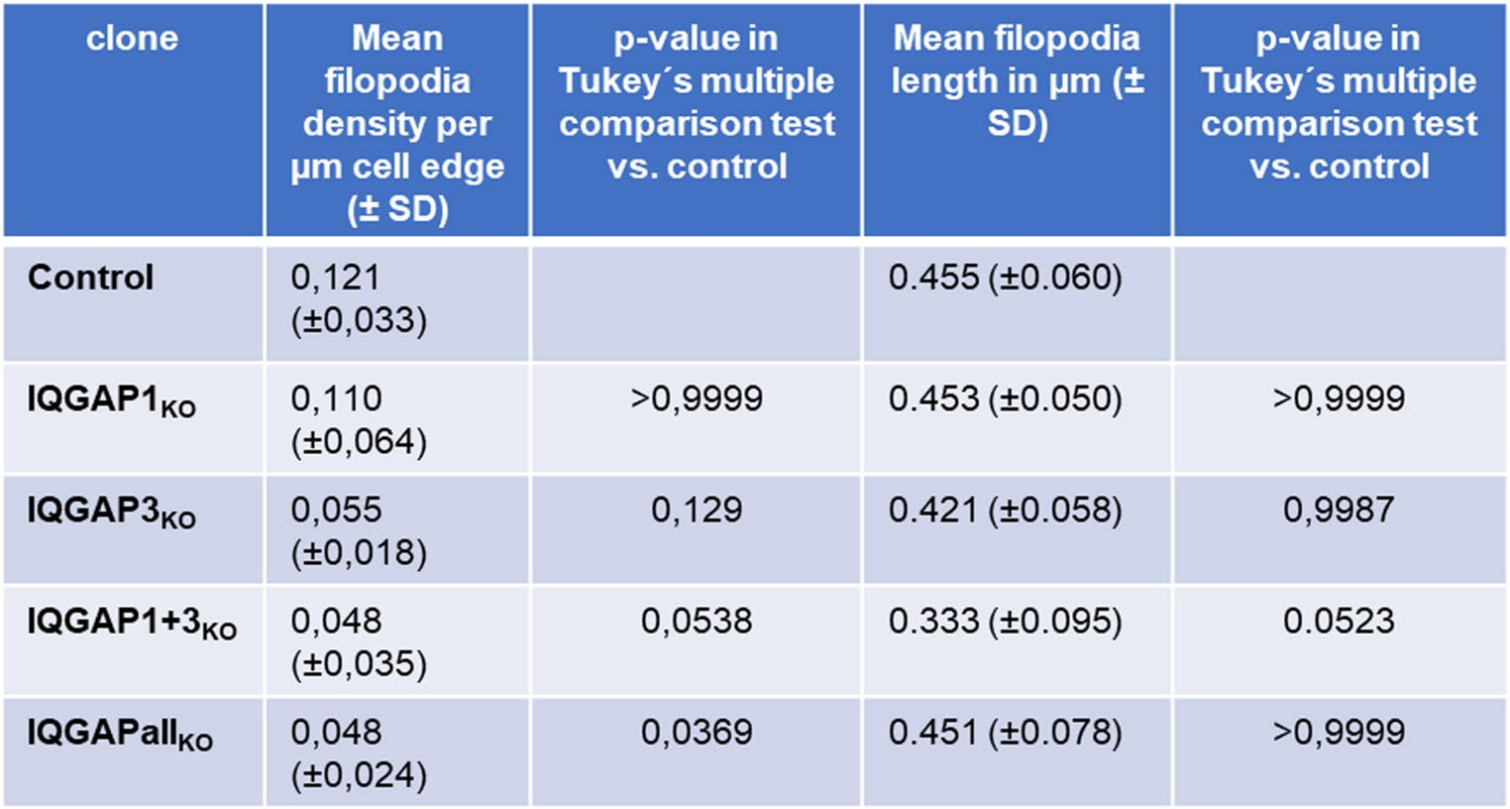
Quantification of filopodia density in IQGAP KO cell clones

Additionally, we quantified the effects of the expression of individual IQGAPs into the IQGAP KO cell clones. Here, the expression of IQGAP1, −2 and − 3, respectively, in the IQGAPall_KO_ cells significantly increased the density of filopodia (supplementary Fig. S2 B). Restoring IQGAP1 in IQGAP1KO cells did not change the density of filopodia (supplementary Fig. S2 B).

Restoring IQGAP3 in IQGAP3_KO_ cell clone showed a significant increase in filopodia density. The same was observed by restoring IQGAP3 in the IQGAP1 + 3_KO_ cell clone. Quantification of the filopodia length upon expression of individual GFP-IQGAPs in the KO cell clones revealed no significant changes.

In summary, expression of individual GFP-IQGAP isoforms into IQGAPall_KO_ cells at least partially restored actin cytoskeleton dynamics of the control cells, suggesting that the observed defects in the KO cells can be attributed to the specific IQGAP KO. This data highlighted the importance of all three IQGAP family members for actin cytoskeleton dynamics and confirmed the functionality of the GFP-IQGAP constructs, consistent with previous reports [27, 32, 58–62].

### IQGAP proteins modulate cellular permissiveness to MARV infection

The IQGAP KO cell clones were further employed to investigate, which steps of the MARV infection cycle were influenced by the absence of single or multiple IQGAP family proteins. We investigated IQGAP KO cell clones for their permissiveness to MARV infection, their capacity to support viral replication/transcription activity, transport of nucleocapsids and release of MARV particles from the cells and spread in cell culture.

We assessed the impact of IQGAP KO on the ability of target cells to support MARV infection. At 24 h post-infection (p.i.), we examined the actin cytoskeleton and the MARV-induced IB formation in fixed cells by immunofluorescence analysis. Actin was detected by phalloidin coupled to Alexa647. The presence of the viral nucleoprotein NP was detected by an anti-NP antibody.

**Fig. 3:**
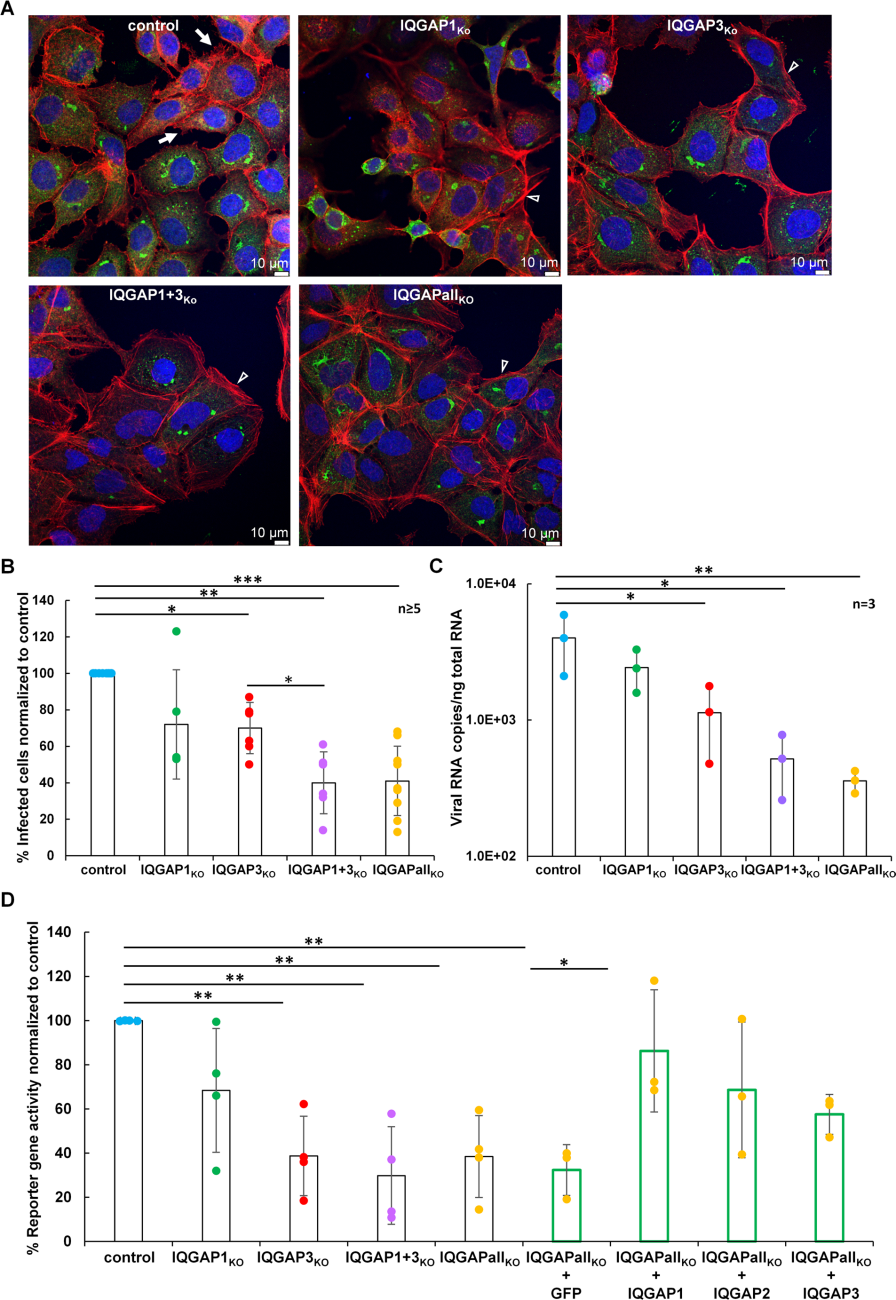
Permissiveness of the Huh-7 KO cell clones to MARV infection. **(A)** Indicated Huh-7 cell clones were infected with MARV at MOI 1, fixed 24 h p.i. and subjected to immunofluorescence analysis. MARV NP was stained with a specific monoclonal antibody and a secondary goat anti-mouse Alexa Fluor 488-conjugated antibody. Actin was stained with Phalloidin-TRITC (shown in red) and cells were subjected to confocal microscopy using a Leica Stellaris microscope. White arrows indicate cell borders with filopodia and arrowheads actin stress fibres. **(B)** Quantification of the permissiveness of Huh-7 KO cell clones to MARV infection. Cells were fixed 18–24 h p.i. for immunostaining of the nucleoprotein and DAPI staining of nuclei. Images were acquired and numbers of NP-positive cells were quantified for each clone. The diagram shows single data points (*n* ≥ 5 experiments) and normalized mean values with the control set to 100%, with indicated standard deviations. Statistical significance was assessed using Tukey´s multiple comparisons test with an adjusted p-value defined as *p* ≤ 0.05 (*), *p* ≤ 0.01 (**), *p* ≤ 0,001 (***). Non-significant differences are not indicated. **(C)** Influence of IQGAP KO in Huh-7 cell clones on MARV transcription and replication. Viral RNA synthesis was quantified by qRT-PCR in MARV infected IQGAP KO and control cells 24 h p.i. and expressed as viral RNA copies per ng of total cellular RNA (*n* = 3). Statistical significance was assessed using Tukey´s multiple comparisons test, with an adjusted p-values defined as follows: *p* ≤ 0.05 (*), *p* ≤ 0.01 (**) and not significant differences are not indicated. **(D)** Luciferase-based MARV minigenome assays were conducted in the indicated IQGAP KO Huh-7 cell clones using Renilla luciferase as a reporter gene. Reporter gene activities were normalized to those of the control cells, which were set to 100%. Data are derived from 3–4 independent experiments and are presented as mean values ± standard deviation. Green bars indicate values from the IQGAPall_KO_ cell clone after back transfection of GFP-tagged IQGAP constructs and GFP as control. Statistical significance was assessed using Tukey´s multiple comparisons test with an adjusted p-value defined as follows *p* ≤ 0.05 (*), *p* ≤ 0.01(**), and nonsignificant differences are not indicated

The analysis revealed that all IQGAP KO cell clones were susceptible to MARV infection (Fig. 3A) and exhibited a similar actin cytoskeleton phenotype as their uninfected counterparts, characterized by an increase in stress fibres and a reduction in the numbers of filopodia (refer to Fig. 2A for comparison). The number of infected cells per microscopic field was counted and normalized to the total number of cells in each field. Compared with control cells, the single IQGAP1 KO cells displayed a non-significant reduction of approximately 30%, whereas IQGAP3 KO cells showed a similar but statistically significant reduction. The double and tripIe KO cell clones (IQGAP1 + 3_KO_ and IQGAPall_KO_) exhibited a significant 60% decrease in the number of infected cells (Fig. 3B).

The effect of the IQGAP KOs was also assessed by determining intracellular viral RNA copies by quantitative real-time PCR (Fig. 3C). A significant reduction of viral RNA copies was observed in IQGAP3_KO_ cells, which was further decreased upon additional KO of IQGAP1 (IQGAP1 + 3_KO_ cells) and IQGAP2 (IQGAPall_KO_ cells) (Fig. 3C).

### Impact of IQGAP1 and IQGAP3 on MARV transcription/replication activities

To assess further the influence of the IQGAP proteins directly on MARV transcription and replication, we determined the activity of the MARV RNA synthesis machinery in the IQGAP KO cell clones using a MARV-specific minigenome assay (Fig. 3D). In this assay, luciferase expression indicates MARV-specific transcription and replication activity [63].

The results of the minigenome assay showed an impact of IQGAPs on MARV transcription/replication. The deletion of IQGAP3 either alone or in combination with IQGAP1 or in the triple KO together with IQGAP2 led to a significant ~ 60% decrease in reporter activity compared to control cells indicating that deletion of IQGAP3 had a strong impact on viral RNA synthesis (Fig. 3D). The IQGAP1_KO_ clone exhibited a non-significant 30% reduction in reporter activity, suggesting that IQGAP1 was not relevant for viral RNA synthesis under these conditions.

However, expression of GFP-IQGAP1 in the IQGAPall_KO_ cell clone resulted in a statistically significant increase in reporter activity. Expressing GFP-IQGAP2 or GFP-IQGAP3 in the IQGAPall_KO_ cells, revealed a positive effect on viral RNA synthesis which was not significant. The expression of GFP had no effect. (Fig. 3D). We confirmed comparable transfection efficiency across the KO cell clones determining the NP expression through western blot analysis using anti-NP antibodies (supplementary Fig. S3 A and B).

### IQGAPs are not required for actin-tail formation at MARV NCs

Because IQGAP1 is known to modulate actin polymerization and dynamics, we further investigated if IQGAP1 is involved in the formation of actin tails observed at one end of the MARV nucleocapsids. To visualize actin tail formation, we infected all IQGAP KO and control Huh-7 cells with recombinant MARV (rMARV-VP30GFP) expressing a fusion protein of VP30 and green fluorescent protein (VP30GFP). VP30GFP, through interaction with NP, fluorescently labeled viral nucleocapsids in the infected cells [15]. Simultaneously, a Tag-RFP-actin encoding expression plasmid was transfected. Live cell imaging revealed actin tail formation at MARV nucleocapsids in the absence of IQGAPs (IQGAPall_KO_) which showed no apparent differences to the control Huh-7 cells (supplementary data Movie 4, 5) demonstrating that IQGAPs are not essential for actin tail formation at MARV nucleocapsids. We had previously shown that MARV NP interacts with Tsg101, which in turn can interact with Alix, a known regulator of actin dynamics [13, 64–66]. It is therefore conceivable that, in the absence of IQGAPs, Tsg101 may recruit actin nucleators to MARV nucleocapsids.

#### Influence of IQGAPs on intracellular transport of MARV nucleocapsids

We next investigated the role of IQGAP family members in modulating the intracellular transport dynamics of MARV nucleocapsids. To this end, we performed time-lapse imaging in various IQGAP KO cell clones infected with rMARV-VP30GFP. Image acquisition was carried out every 500 milliseconds over a period of 100 s (see supplementray data Movies 6, 7, 8, 9, 10).

**Fig. 4:**
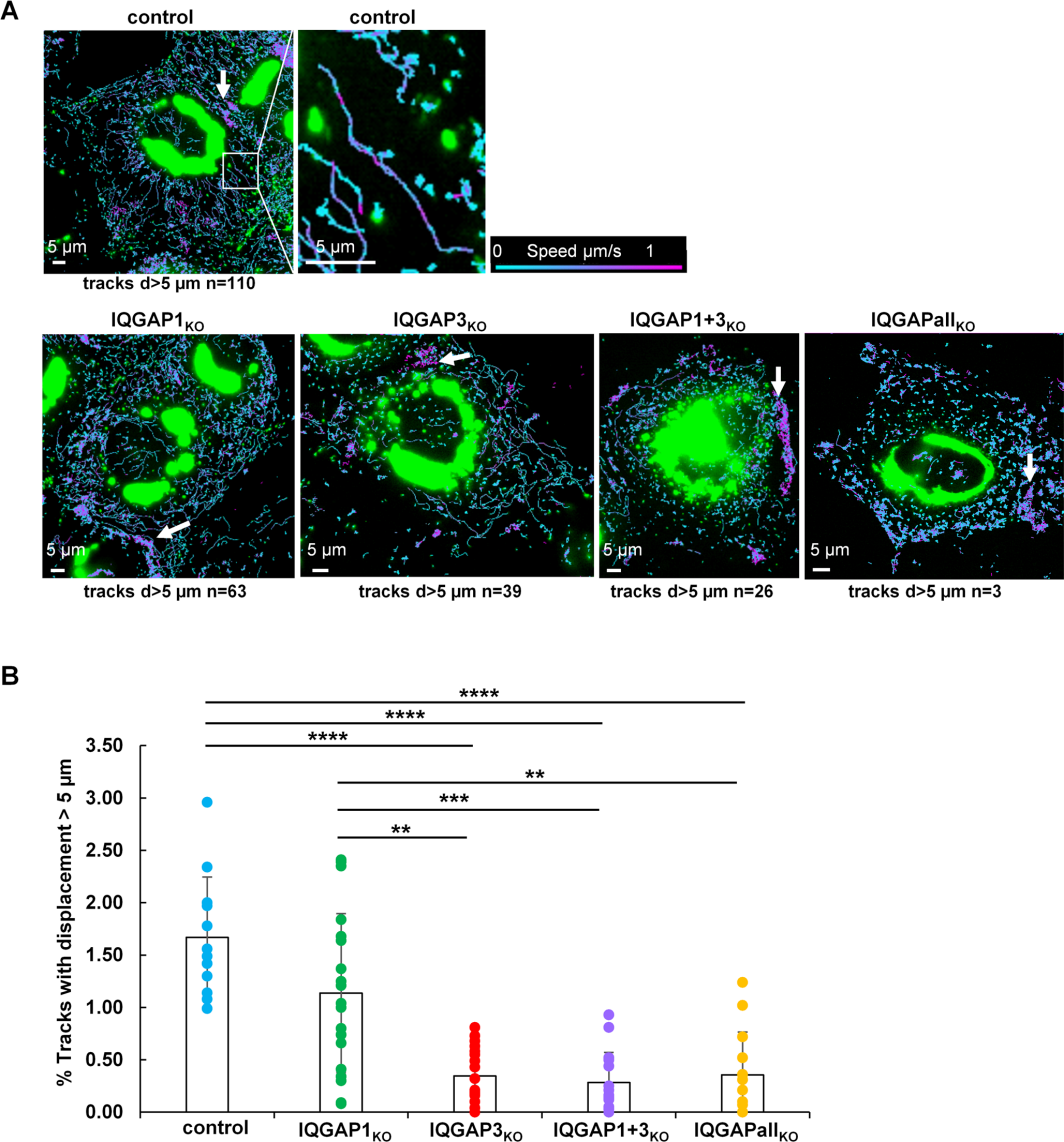
Impact of IQGAP knockout on intracellular transport dynamics of MARV nucleocapsids. Huh-7 IQGAP KO and control cells were infected with a recombinant MARV encoding VP30-GFP to visualize nucleocapsids. Live-cell imaging was performed at 48 h h p.i. using a Leica DMI6000 microscope. Time-lapse image sequences were acquired at 500 ms intervals over a period of 100 s. For each clone, two independent experiments were conducted, capturing a minimum of 14 cells and 27,000 trajectories. Particle tracking was carried out using the IMARIS Spots™ function. **A**. Representative nucleocapsid trajectories are shown with color coding from cyan to magenta, indicating increasing velocity from 0 to 1 μm/s. White arrows highlight fast-moving nucleocapsids at the cell periphery (magenta, approximately 1 μm/s). The amount (n) of trajectories with a displacement (d) of more than 5 μm is indicated per shown example for each cell clone. **B.** Quantitative analysis of nucleocapsid transport: bar graph with plotted single data points displays the mean values ± standard deviation in percentage of trajectories with displacements greater than 5 μm. Statistical significance was assessed using Tukey´s multiple comparisons test with an adjusted p-value defined as follows *p* ≤ 0.01 (**), *p* ≤ 0,001(***), *p* ≤ 0,0001 (****). Non-significant differences are not indicated

Previous live-cell imaging studies have demonstrated that MARV nucleocapsids predominantly exhibit short-distance, oscillatory movements reminiscent of Brownian motion, with only a minor subset undergoing long-range, directed transport exceeding 10 μm [48]. To quantify transport parameters in our system, we analyzed several thousand nucleocapsid trajectories per cell clone using IMARIS image analysis software. Analysis of long, straight trajectories revealed that the nucleocapsids moved with varying velocities over the course of their travel (Fig. 4A, boxed area), potentially corresponding to actin-mediated pulsatile events as previously described [47].

A displacement of more than 5 μm may suffice to translocate nucleocapsids from the perinuclear IBs, where they are formed [15], to the plasma membrane, where budding takes place. Therefore, we quantified the number of trajectories exceeding displacement of 5 μm across different IQGAP KO clones (Fig. 4B). In control cells, 1.62% of trajectories exhibited displacements greater than 5 μm. This fraction was significantly decreased in all IQGAP KO cell clones except in IQGAP1_KO_ cells (Fig. 4B).

Additionally, we observed regions within infected cells that contained numerous fast-moving nucleocapsids, predominantly located at the cell periphery (see Fig. 4A, white arrows). Despite their rapid motion, these nucleocapsids exhibited minimal net displacement. As in these peripheral regions the nucleocapsids often appear as dot-like structures it is possible that they assume a perpendicular orientation relative to the imaging plane. One possibility is that these nucleocapsids are localized within plasma membrane protrusions - such as filopodia or microspikes - whose distal tips are not anchored to the substrate. The underlying mechanism responsible for these rapid, non-directional movements remains unclear and need further studies to understand the origin of these phenomena.

### IQGAP1 and IQGAP3 together support MARV release

**Fig. 5:**
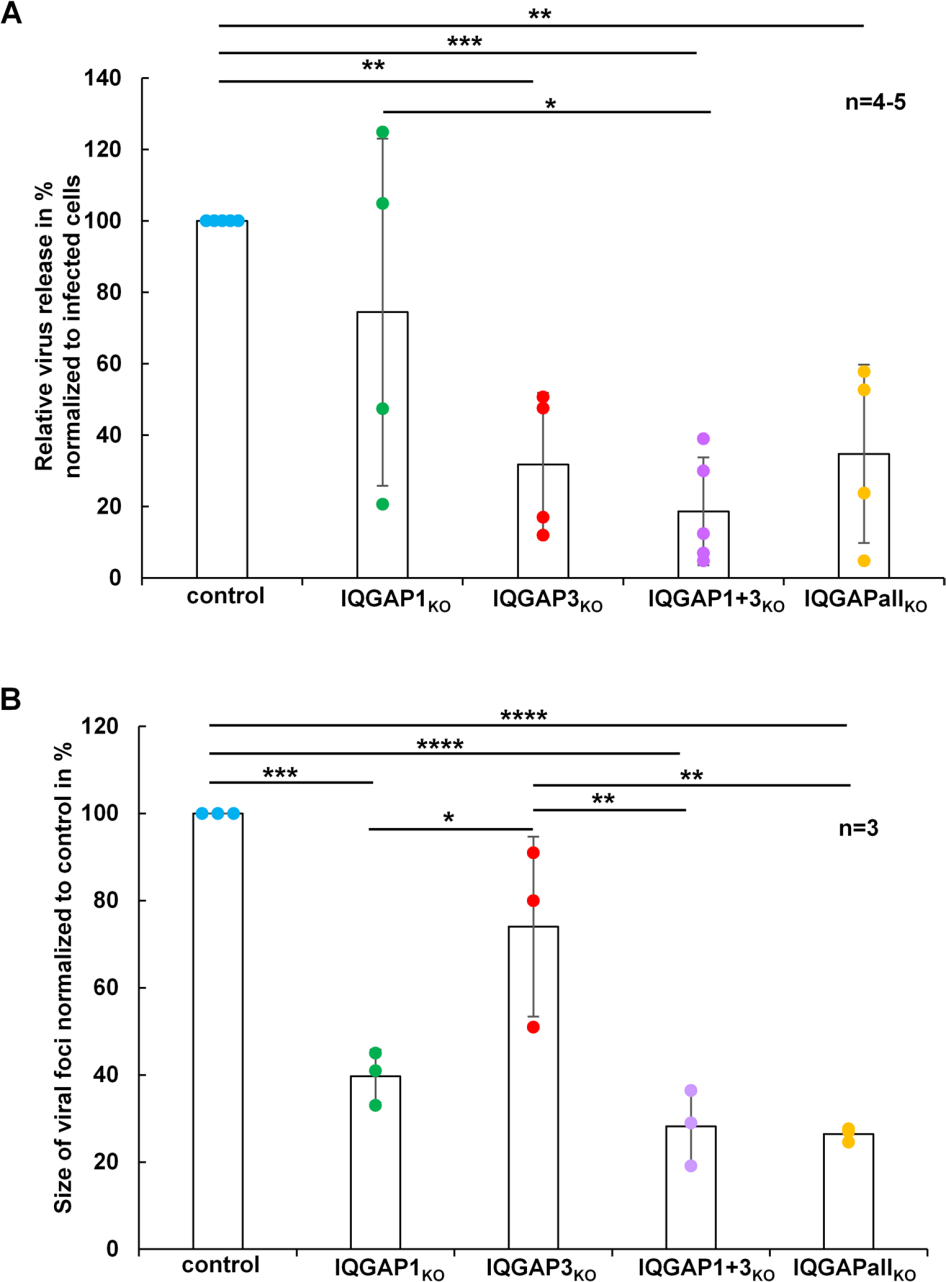
Influence of IQGAP KO in Huh-7 cell clones on MARV release and plaque size.** A. **IQGAP KO cell clones were inoculated with equal amounts of infectious MARV (MOI 0.1) and culture supernatants collected for TCID50 titration at 24 h p.i. The amount of infected cells was determined by immunofluorescence staining of the nucleoprotein. The diagram shows relative virus release to control cells normalized to the number of infected cells from four independent experiments with standard deviations. **B.** IQGAP KO cell clones were inoculated with MOI of 0.1 pfu/cell recombinant MARV expressing VP30-GFP and foci formation was monitored at three days p.i. Mean focus size (>100 foci) of control cells was set to 100% and compared to KO cell clones. Diagram shows mean values from 3 independent experiments of n≥49 foci per KO cell clone with standard deviations. Statistical significance was assessed using Tukey´s multiple comparisons test with an adjusted p-value defined as follows p≤0.01 (**), p≤0,001(***), p≤0,0001 (****) and non-significant differences are not displayed.

We then quantified the release of infectious MARV from the IQGAP KO cells at 24 h p.i. by TCID50 titration of the supernatants. We observed a non-significant reduction of virus release to approximately 30% due to IQGAP1 KO. In contrast, a significant reduction of 65% was detected in IQGAP3_KO_ cells, 80% in IQGAP1 + 3_KO_ cells, and 70% in IQGAPall_KO_ cells (Fig. 5A).

Analysis of the supernatants by western blot using MARV NP- and VP40-specific antibodies confirmed the TCID50 assay results, showing significant decrease in NP release from all IQGAP KO cell clones (supplementary Fig. S3 C-D). VP40 levels in the supernatants were also significantly reduced in IQGAP3_KO,_ IQGAP1 + 3_KO_ and IQGAPall_KO_ cell clones (supplementary Fig. S3 C-D).

Together, these results suggested that simultaneous KO of IQGAP1 and IQGAP3 leads to an additive reduction in MARV release.

#### IQGAP1 and IQGAP3 support MARV cell-to-cell spread

To assess whether IQGAP knockouts directly influenced MARV spread in cell culture, we conducted a focus forming assay using a VP30-GFP expressing recombinant MARV. After infection, the cells were overlaid with Avicel™, and the resulting focus sizes were measured at three days p.i. (Fig. 5B). Compared to the control cells, the KO of IQGAP1 led to a reduction in foci sizes by 61% and the KO of IQGAP3 resulted in a 27% reduction. The combined KO of IQGAP1 and IQGAP3, as well as the triple KO involving IQGAP2, resulted in a similar reduction in foci size: approximately 70%. These findings suggested that IQGAP1 exerts a more significant impact on the spread of MARV particles than IQGAP3, and together, both proteins play important roles for MARV dissemination from cell-to-cell.

## Discussion

Our results suggest that among the IQGAP family members, IQGAP1 and IQGAP3 represent the major pro-viral factors, which supported MARV propagation at several steps during the replication cycle (Table 3).

**Table 3.**
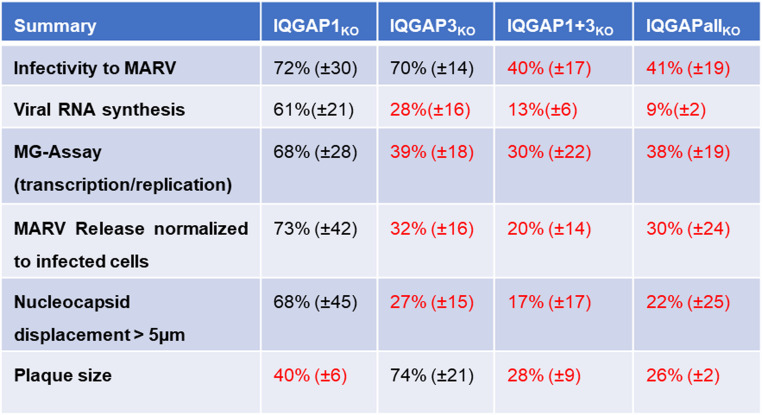
Tabular summary

Data is displayed as mean values normalized to the control Huh-7 cell clone

We found that both, KO of IQGAP1 and IQGAP3 reduced the permissiveness to MARV infection (Fig. 3). Filovirus uptake into cells occurs via actin-dependent endocytic pathways such as a macropinocytosis-like uptake [67, 68]. The involvement of IQGAP1 in the regulation of macropinocytosis has been demonstrated by several publications [69–73]. Thus, the presented data cannot exclude that IQGAP family members are involved in the endocytosis of MARV. This has to be explored in further studies.

The reduction of MARV-infected cells in the IQGAP KO clones could also have been caused by an impact on MARV transcription/replication. Here we could show that viral RNA synthesis efficiency is mainly hampered by IQGAP3 KO (Fig. 3C -D). This effect is independent of IQGAPs possibly impacting the endocytosis of MARV because the minigenome assay used is based on transfecting plasmids expressing the viral proteins (Fig. 3D). IQGAP3 has been suggested to be important for proper cell-cycle progression into the S-phase and cell proliferation [74, 75]. It is conceivable that MARV uses cell cycle dependent host cell factors, like nucleosides, cyclin-dependent kinases or other enzymes for efficient RNA synthesis as shown for other viruses [76]. Therefore, IQGAP3 deficiency could restrict access to those factors. In addition, because IQGAP3 is recruited into IBs like all other IQGAPs, it is possible that the supportive effect on transcription/replication could be the result of direct effects on the viral replication/transcription complex. This assumption also needs further research.

All three IQGAP isoforms were detected in MARV IBs and at one end of the moving nucleocapsids in the cytosol outside the IBs (Fig. 1 and supplementary movies 1, 2, 3).

Regulation of actin polymerization by IQGAP1 is well documented [23, 42, 77–80]. Given that the transport of MARV nucleocapsids is actin- and Arp2/3-dependent, IQGAP1 could play a crucial role in the regulation of actin-tail formation at one end of the nucleocapsids [15, 49]. Although the presence of IQGAPs is not obligatory for actin tail formation and movement of nucleocapsids (supplementary movie 5), it might regulate the efficiency of nucleocapsid transport dynamics. IQGAP1 could directly influence the actin turn-over at the growing plus-end of actin filaments involved in pushing the nucleocapsids for efficient displacement [80]. In addition, IQGAPs may recruit effectors of actin polymerization such as Rac1 or Cdc42, to MARV nucleocapsids, which are essential for the nucleocapsids’ efficient directed transport [81]. This idea is supported by our recent data showing that Rac1 and WAVE1 knockdown by siRNA affected the straightness of EBOV nucleocapsid movement [48]. Baculovirus nucleocapsids are propelled by Arp2/3-induced actin filament branching, which leads to the formation of actin-tails. This process is activated via the interaction of Arp2/3 with the viral capsid protein p73/83 [82]. A similar mechanism could be hypothesized for MARV, however, it is currently unknown, how the actin-tail formation is directed to MARV nucleocapsids and especially to one end of the particles [15].

After MARV nucleocapsids have managed the transport through the cytosol and finally arrived at the cell surface, they are enveloped by budding through the plasma membrane and *de novo* virus particles are released from the cells (reviewed in [13, 14]. Our data demonstrated that the double KO of IQGAP3 and IQGAP1 has a pronounced impact on the release of infectious MARV (Fig. 5A). Filopodia are the primary sites of MARV budding [49, 83, 84] and IQGAP1 has been shown to play an important role in the formation of filopodia by the recruitment of Cdc42, Arp2/3 and formins [85, 86]. It is therefore possible that the mediation of filopodia-formation by IQGAP1 supports the MARV budding and spread as we have shown that mainly IQGAP1 KO in particular reduced the plaque size in cell culture (Fig. 5B). In line with these results, a recent study demonstrated the importance of Cdc42-IQGAP1-Arp2/3 axis in HIV spread [87]. A supportive role of IQGAP1 in virus release has also been shown for other enveloped viruses such as Moloney murine leukemia virus [88], classical swine fever virus [89] and EBOV [90]. Future studies have to show whether IQGAP2 and − 3 also affect the replication cycle of other MARV isolates and other filoviruses.

In summary, our results indicate the involvement of IQGAP1 and IQGAP3 at several steps of the MARV replication cycle (Table 3). IQGAP3 was shown to be the main player among the IQGAPs to support MARV transcription and replication, as well as release of infectious MARV into the cells supernatant. The function of IQGAP1 in regulation of actin dynamics seems to be required for the spread of MARV particles from cell-to-cell. Our earlier observation that transcription of IQGAP1 and IQGAP3 is upregulated at 23 h p.i. in Huh-7 cells suggests that MARV actively promotes the upregulation of these pro-viral factors and supports the present data that both proteins enhance MARV propagation [91]. Further investigations into the precise interactions of IQGAPs with MARV proteins, and how MARV specifically upregulates IQGAPs and IQGAP-interacting proteins will shed new light on the transport process of nucleocapsids and may help to uncover new antiviral targets.

## Supplementary Information

Below is the link to the electronic supplementary material.

Supplementary Movie 1

Supplementary Movie 2

Supplementary Movie 3

Supplementray Movie 4

Supplementray Movie 5

Supplementray Movie 6

Supplementary figure 6 Supplementary Fig. S3

Fig. S1

Supplementary Movie 7

Supplementary Movie 8

Supplementary Movie 9

Supplementary Movie 10

Supplementary figure 7 Supplementary Fig. S1

High Resolution Image (TIFF 3.65 MB)

Supplementary figure 8 Supplementary Material 14(PNG 467 KB)

Supplementary Fig. S2

## Data Availability

The supplementary videos will be provided to the public under the Marburg University research data repository. https://data.uni-marburg.de/communities/373063b0-b1e1-45dc-b8f4-3278d82cc88c/browse/faculty?value=FB20:Medizin.
