# Supplementary figures and images for: IQGAP1 and IQGAP3 are critical host factors for Marburg virus replication, nucleocapsid transport, and cell-to-cell spread
Source: Cell Mol Life Sci. 2026 Feb 9;83(1):101. doi: 10.1007/s00018-025-06047-3 (PMC12886712; doi:10.1007/s00018-025-06047-3)

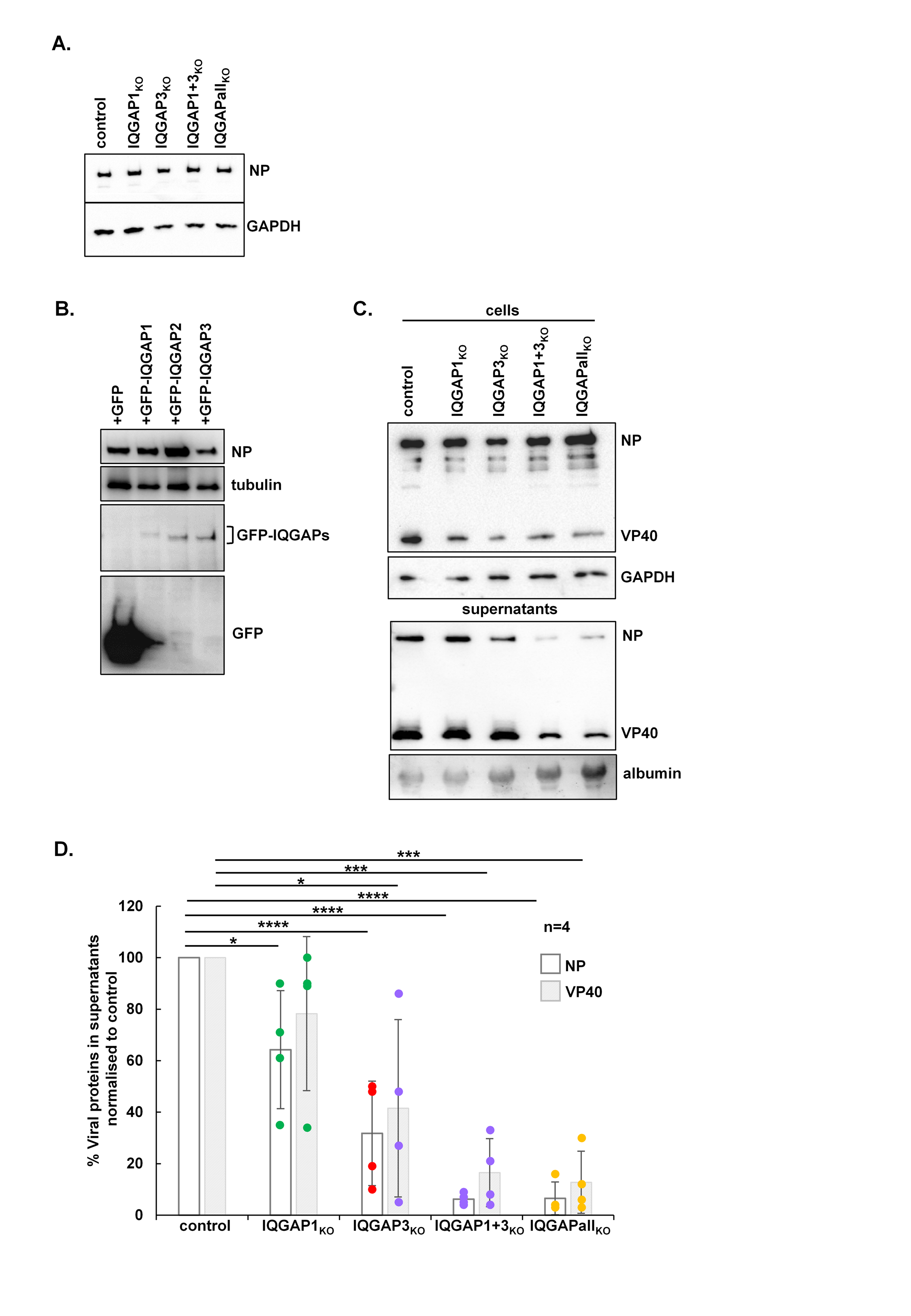

Supplement: Supplementary file 7 — Supplementary Fig. S3 [file 18_2025_6047_Fig6_ESM.png]

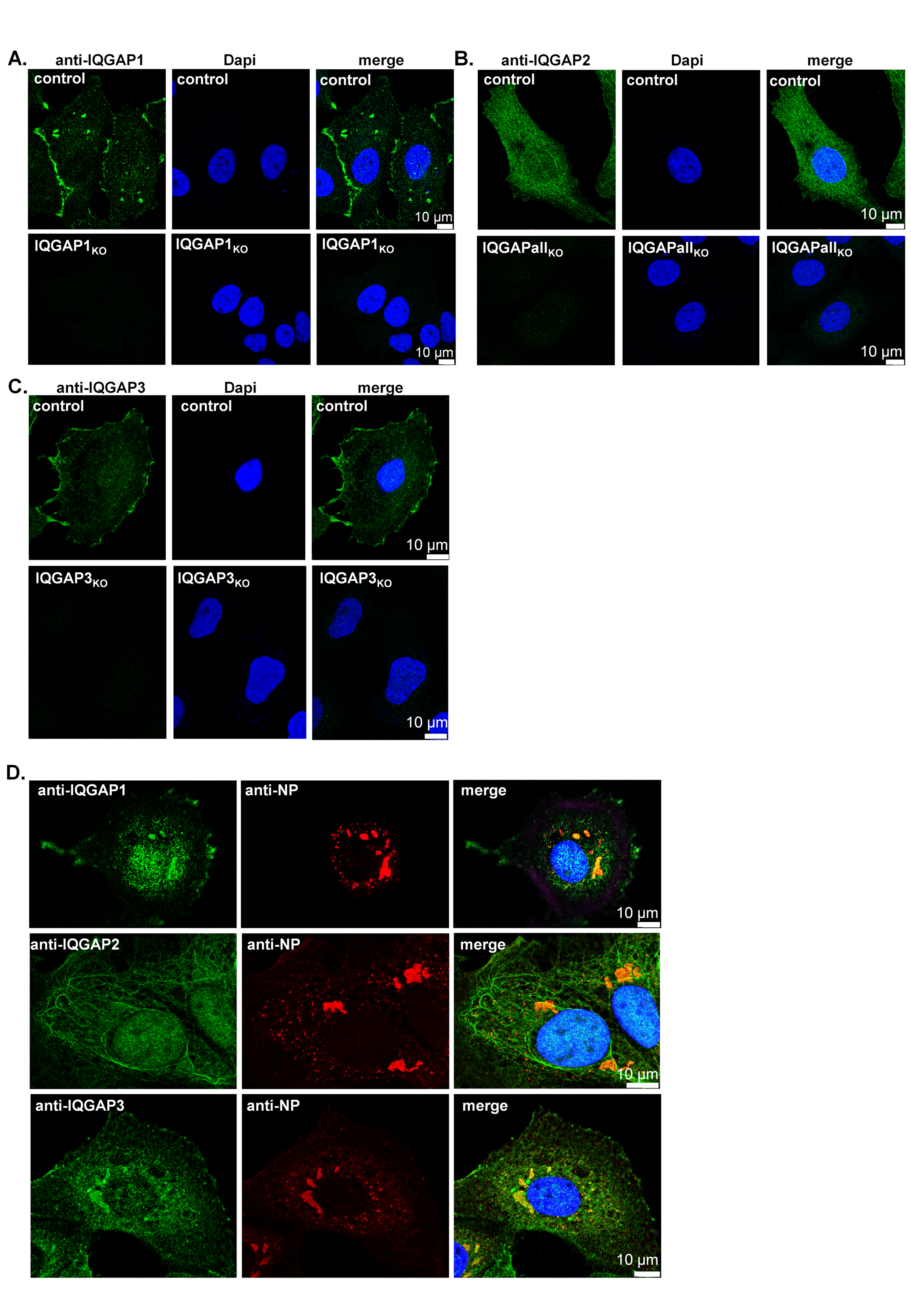

Supplement: Supplementary file 13 — Supplementary Fig. S1 [file 18_2025_6047_Fig7_ESM.png]

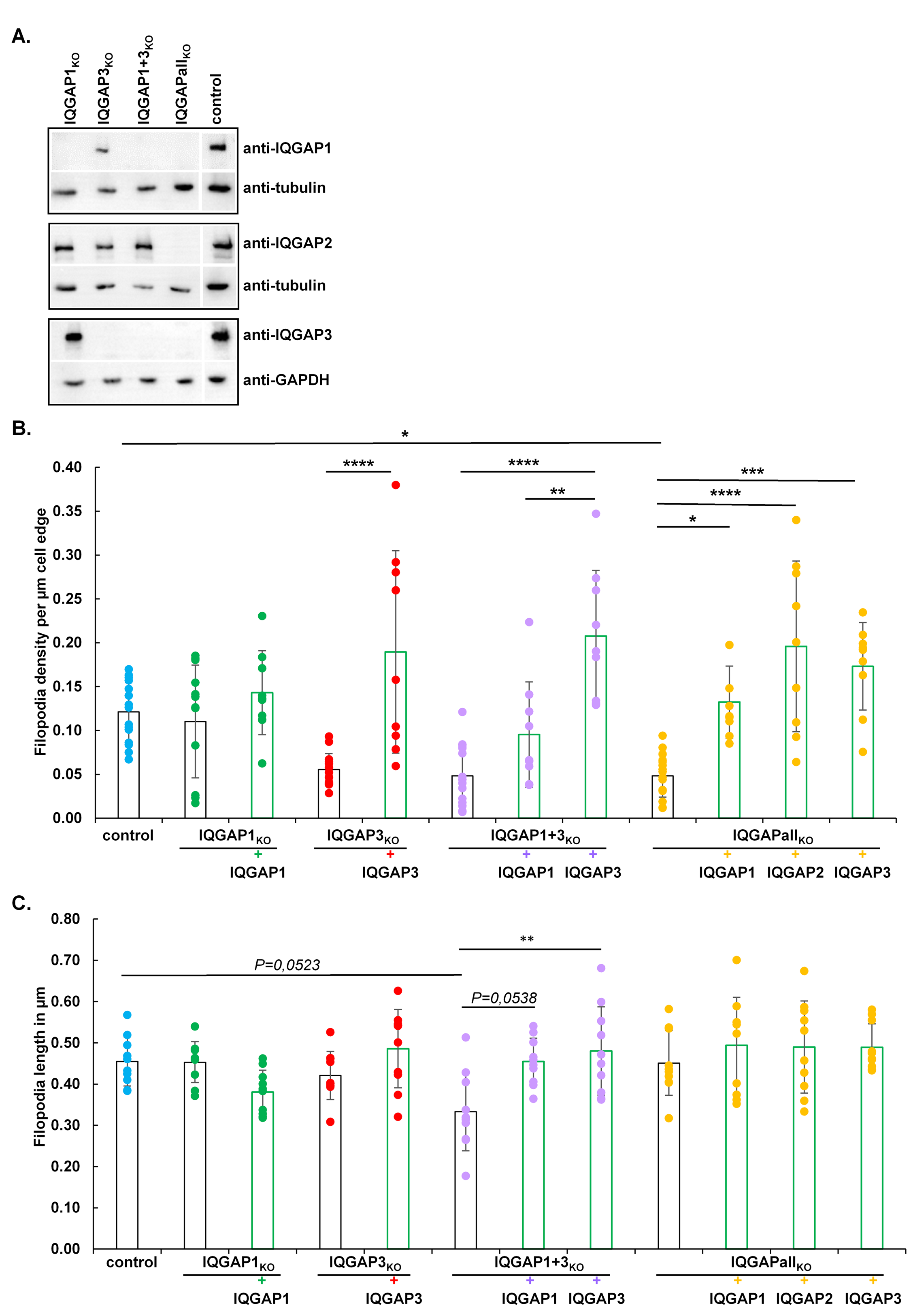

Supplement: Supplementary file 15 — Supplementary Material 14(PNG 467 KB) [file 18_2025_6047_Fig8_ESM.png]
